# Player-Reported Perceptions of Lameness Risks and Contributing Factors for Polo Horses: Results from a Survey

**DOI:** 10.3390/ani15213136

**Published:** 2025-10-29

**Authors:** Anton Schumacher, Roswitha Merle, Sabita Stöckle, Heidrun Gehlen

**Affiliations:** 1 Equine Clinic, Freie Universität Berlin, Oertzenweg 19b, 14193 Berlin, Germany; anton.schumacher@fu-berlin.de (A.S.);; 2 Institute of Veterinary Epidemiology and Biostatistics, Veterinary Centre for Resistance Research, Freie Universität Berlin, 14163 Berlin, Germany

**Keywords:** polo, horse, health

## Abstract

**Simple Summary:**

This study examined the health risks faced by polo horses by analysing the responses of 145 polo players to an international online survey. The focus was on how players perceive the risk of lameness and the factors that may influence its occurrence. The results revealed that the risk of lameness increased significantly with the horses’ age and varied between breeds. In contrast, factors relating to the players, such as their age, sex, playing experience and how they kept their horses, showed no significant effect. Interestingly, the perception of risk was only linked to the players’ handicap level: more experienced players with higher handicaps tended to rate the likelihood of injuries as lower. The most cited cause of injury was poor ground conditions, emphasising the importance of suitable playing surfaces for the welfare of horses. These insights emphasize the necessity of targeted preventive strategies and further research to enhance the health and wellbeing of polo horses.

**Abstract:**

Polo is an equestrian sport with a long tradition, but to date there is little scientific data on health risks, particularly lameness. In an international online survey, 145 polo players (47.6% female, 52.4% male) with an average of 13.4 years of playing experience were questioned about lameness risks and influencing factors. The respondents reported a median of six horses, most frequently mares as their best-performing and healthiest animals. Horse age was significantly associated with the risk of lameness, increasing by approximately 19.5% per year (OR = 1.195; *p* = 0.030) and differed depending on the breed of horse. Player-related variables such as age, sex, level of experience and type of husbandry had no significant influence. Risk perception was only associated with the handicap: players with a higher handicap rated the risk of injury as lower. Sex, age and horse ownership were not relevant. Unfavourable ground conditions were cited as the most common cause of injury (58.6%). The results show that players believe key predictors of lameness, in particular the age and breed of the horses, are objective factors, while subjective risk assessments are primarily influenced by the players’ level of experience. Further studies are necessary to improve horse health in polo and to enable preventive measures.

## 1. Introduction

Polo is considered the oldest equestrian sport in the world with increasing popularity [1,2,3]. In this team sport, two teams of four players each compete. A game is divided into several periods called chukkers. The number of chukkers varies depending on the tournament and its level of competition. Typically, four chukkers are played, but for higher-handicap matches and more prestigious tournaments, the number can increase to eight, which occurs only in particularly elite final matches.

Nowadays, as awareness of animal health and welfare increases, scientific studies on health risks in polo remain scarce, particularly those that systematically assess potential stressors and risk factors affecting polo horses [4,5,6,7,8,9].

Most studies have been based on biomechanical analyses investigating various aspects of polo performance, such as athletic capacity and equine movement patterns using GPS data [3,9,10,11,12]. In addition, an internet-based survey explored the perspectives of players and trainers. Although this approach yielded valuable insights, it was subject to selection, distribution, and response bias [4].

Detailed information on the prevention of injury is unavailable. Surveys investigating active players’ perceptions of lameness risk remain scarce. Targeted and systematic research in this area is therefore urgently needed to support the long-term health and welfare of polo horses [11,13]. A notable study by Inness and Morgan found an injury rate of 10.6% per season among polo horses. This study also examined the risk of lameness in detail. They found that superficial flexor tendon injuries were the most frequently reported type of injury in polo horses [4]. Tendon and ligament injuries in polo are often the result of repetitive strain, affecting not only the superficial flexor tendon, but also the collateral ligaments of the distal interphalangeal joints. Polo horses over nine years of age are at an increased risk of developing diseases that affect these collateral ligaments [14]. Additionally, joint diseases, particularly osteoarthritis, are a common cause of lameness in polo horses. Osteoarthritis of the fetlock joint is considered the main reason for early retirement [15].

With the increasing professionalization of polo, the awareness of horse enthusiasts regarding animal welfare is also growing, which should encourage organizers and governing bodies to promote transparency and place greater emphasis on animal welfare [16]. Therefore, the perspectives of polo players on injury risks of their horses are becoming increasingly relevant—particularly regarding possible correlations with players’ age, sex, or level of experience, especially because more experienced players perceive the risk of falls and injuries for the rider in polo as lower [1].

In polo, the handicap serves as a measure of experience and is therefore considered a potentially influential factor in risk assessment. Handicaps in polo range from −2 for beginners to +10, a level attained by only a handful of elite athletes worldwide [13].

Research in polo is further complicated by the sport’s decentralized structure. The large number of independent associations, often lacking an overarching regulatory body, hinders the systematic collection of health data and makes large-scale studies more challenging [2,17]. Similar structural challenges also were identified in other equestrian disciplines, such as dressage and show jumping [18,19]. However, experience from other fields, such as human medicine, shows that close collaboration between professional associations and researchers can be crucial for addressing knowledge gaps and establishing health related priorities more effectively [20,21]. Therefore, the present study aimed to systematically analyse both health-related and subjective assessments provided by active polo players [4]. The primary focus was on identifying factors associated with the occurrence of lameness in polo horses. In addition, this study examined how players perceive general injury risks in polo and to what extent individual characteristics of the player, such as age, sex, or handicap, influence this perception. Potential sex-related differences in horses regarding the assessment of their performance, stamina, and ambition were also considered. Ambition in polo horses is reflected in their motivation and willingness to actively participate in the game and to carry out tasks with commitment. An ambitious polo horse responds attentively to commands and game situations, shows initiative, remains focused even under stress, and uses its energy purposefully to perform successfully without undermining the rider’s control. In addition, it actively engages in physical contact with other horses and strives to be faster. The goal was to achieve a distinct understanding of factors related to the perceived risk of lameness in polo ponies and to use this as a foundation for future research and communication with associations and sports organizations.

## 2. Materials and Methods

An internet-based questionnaire was developed to collect data and was made available via the LimeSurvey platform. This was the open-source version of LimeSurvey (Version 5.4.2, LimeSurvey GmbH, Hamburg, Germany; https://www.limesurvey.org, accessed on 26 August 2024), hosted on the servers of Freie Universität Berlin, Germany. The questionnaire, entitled “Survey on Polo Horses”, comprised a total of 62 questions, divided into four thematic sections: general information, additional questions, questions for breeders, and horse-specific information. To enable the broadest possible international participation, the survey was offered in German, English, and Spanish. Among other things, the questionnaire collected information on horse health, husbandry, training, and injury prevention measures, as well as the respondents’ playing experience. Demographic data of the players, including age, sex, and country of origin, were also collected.

Only active polo players were eligible to participate. The questionnaire was developed based on the practical and scientific experience of the authors and was tested in a pilot phase with seven experts in polo and equine medicine. Feedback from four veterinarians, the German national coach, and two professional polo players led to adjustments to the content and language of the questionnaire.

Data were collected between 14 October 2022, and 26 August 2024. The questionnaire was distributed at polo tournaments in Germany and Argentina with an international player lineup. The tournaments ranged from the lowest to the highest playing level. While the survey was completed together with the players at the tournaments, it was also distributed via relevant social media channels. Access was provided via an invitation link.

The survey was conducted completely anonymously. Only responses from participants who indicated that they were actively involved in polo were included in the analysis.

For statistical analysis, descriptive methods were used to summarize the characteristics of the sample. The mean, median, and interquartile range (IQR) were calculated for continuous variables. Binary logistic regression analysis was performed to investigate possible factors influencing the occurrence of lameness in polo horses. The dependent variable was lameness as reported by the players (coded as “yes” = 1, “no” = 0). The regression model included both continuous variables (“polo player since… in years” and “age of horses”) and categorical variables: player gender (“male” or “female”), player age (“under 30 years” or “over 30 years”), stabling type (“extended stabling” or “year-round free stabling”), horse breed (categories: “Polo Argentino,” “Polo Argentino/Purebred,” and “Thoroughbred”), and horse’s sex (“male” or “female”).

In the context stabling, ‘extended stabling’ refers to any method of horse management where horses are kept in stalls or shelters, with the option of additional turnout or paddock access. In contrast, ‘year-round free stabling’ describes a management system in which horses are kept on pasture permanently throughout the year. Regarding the variable ‘horse breed’, this refers to the breed affiliation of the polo horses. ‘Polo Argentino’ is an open stud line specifically bred for polo. The “Polo Argentino/Thoroughbred” category includes crosses between classical racehorses (Thoroughbreds) and polo horses, while “Thoroughbred” refers to purebred racehorses.

Three simple ordinal logistic regression analyses with a logit link were conducted to investigate potential associations between horse’s sex and three performance dimensions: willingness to perform, ambition during play, and endurance during play. In all three models, horse’s sex was included as an independent variable. The dependent variables were grouped into three levels (“very high,” “high,” “partly/partly”); the categories “low” and “very low” were not selected and were therefore excluded. The response options were not further defined by examples or concrete descriptions of the horses’ characteristics.

In addition, a univariate analysis of variance was carried out to investigate the relationship between the estimated risk of injury for horses in polo (dependent variable) and various demographic and sport-related characteristics of the players (independent variables). The dependent variable “How do you rate the risk of injury for horses in polo?” was divided into four ordinal scaled levels (“no risk”, “low risk”, “increased risk”, “high risk”) and numerically coded for analysis. The continuous characteristics handicap (value range: −2 to +10) and number of horses owned as well as the categorical characteristics gender of the player (“female” or ‘male’) and age of the player (“under 30 years” or “over 30 years”) were included in the model as independent variables. The data basis for this was 145 valid cases. To check the requirements for the variance analysis, a visual inspection of the residual distribution was carried out using a histogram. This revealed no serious deviations from the normal distribution.

Statistical analyses were performed using IBM SPSS Statistics software (version 29.0.2.0). Nagelkerke’s R^2^ was used to assess model quality. Regression coefficients (B), their standard errors, *p*-values, and—where applicable—odds ratios (OR) with 95% confidence intervals were used for interpretation. A significance level of *p* < 0.05 was applied to all inferential statistical tests.

## 3. Results

### 3.1. Descriptive Analysis

In this survey, the questionnaires of a total of 145 people were analyzed. Data sets from participants who abandoned the questionnaire after only a few questions were excluded from the analysis. The sample consisted almost equally of 47.6% female and 52.4% male participants. The age distribution showed that most respondents were over 30 years old.

The average polo experience was 13.4 years. The mean handicap of the participants was 0.28 on a scale from −2 to +10, indicating an overall low to medium playing ability. These key figures showed a wide spread around the mean with a slightly left-skewed distribution. The interquartile range was between 4.0 and 17.8 years.

Regarding horse ownership, the respondents reported a median of 6 horses, ranging from 0 to 101 horses, indicating a high degree of variability.

A clear pattern emerged in the assessment of their best-performing polo horses: 74.8% classified their best-performing horse as female, while 61.8% stated that their healthiest horse was also female.

The participants were divided on the issue of changing horses during a chukker, but only 25.4% favored not changing horses at all during the chukker.

Regarding the current chukker duration of 7:30 min, most respondents rated it as “acceptable” to “very good.” The most common cause of injuries to polo horses, cited by 58.6% of participants, was poor ground conditions. Other frequently cited causes were collisions between horses or players (24.1%), being hit by a ball or mallet (20%), and falls (14.5%).

The assessment of the risk of injury for horses in polo was predominantly critical: 56.9% rated the risk as “increased,” and 15.4% as “high.” 26.9% rated it as “low,” while only 0.8% saw no risk of injury.

**Figure 1:**
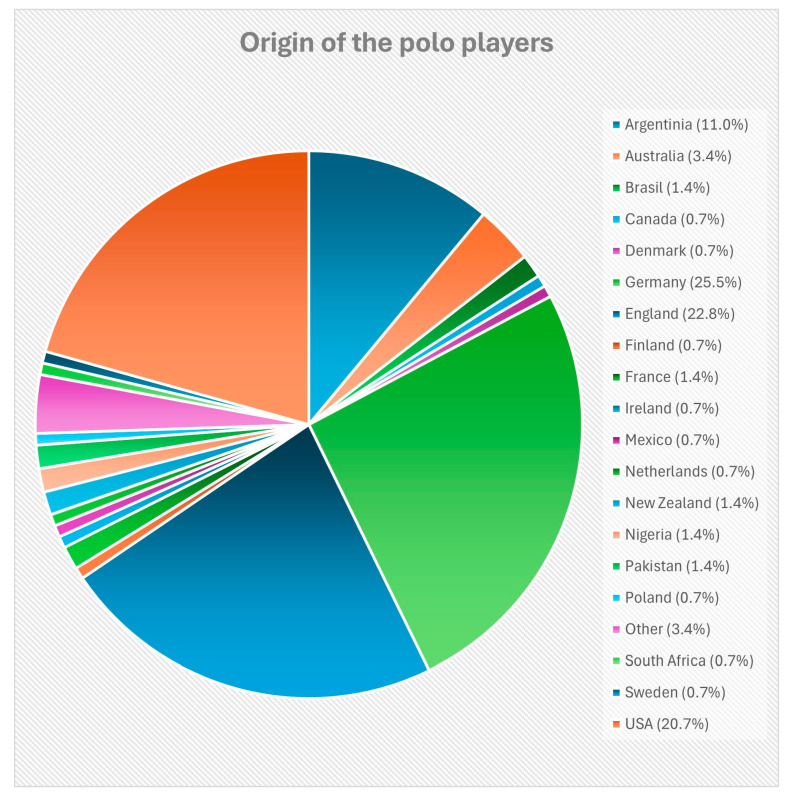
Distribution of the countries of origin of the survey participants (*n* = 145). The percentages of the countries from which the surveyed polo players come are shown.

Responses to breeder-related questions were excluded from the analysis due to insufficient case numbers (Figure 1) (Table 1).

### 3.2. Factors Associated with Player-Reported Lameness

This study analysed various factors influencing the risk of lameness in polo horses. First, the characteristics of the players—playing experience, gender and age—showed no significant correlation with the occurrence of lameness (playing experience: OR = 1.061; *p* = 0.152; gender: OR = 2.779; *p* = 0.253; age: OR = 0.275; *p* = 0.089).

The type of housing for the horses (e.g., open stables) was also not associated with an increased risk of lameness (OR = 1.106; *p* = 0.913).

In contrast, the differences between horse breeds were more pronounced: Crossbreeds of Polo Argentino horses and Thoroughbreds had a significantly increased risk of lameness, which was higher than that of Polo Argentino horses (OR = 9.955; 95% CI: 1.160–85.466; *p* = 0.036). However, the effect was not significant for thoroughbreds alone (OR = 2.507; *p* = 0.316).

The age of the horses, on the other hand, proved to be a relevant factor: with each additional year of life, the risk of lameness increased by approximately 19.5% (OR = 1.195; *p* = 0.030; see Table 2).

### 3.3. Sex Effects on Performance Scores

The ordinal regression showed a significant increase in the odds for “high” (OR = 35.15, 95% CI: 10.09–122.29, *p* < 0.001) and a moderately increased effect for “very high” (OR = 2.43, 95% CI: 1.33–4.43, *p* = 0.004) compared to the reference (“partly/partly”). Male horses showed a non-significant trend toward higher odds (OR = 2.52, 95% CI: 0.94–6.75, *p* = 0.067), with Nagelkerke’s R^2^ of 0.055 indicating a possible relevance of horse’s sex. Higher odds here mean that male horses tended to be rated more frequently with a higher willingness to perform.

For ambition in play, there was a highly significant increase in the odds for “high” (OR = 13.45, 95% CI: 5.41–33.42, *p* < 0.001), while “very high” showed no difference from the reference (OR = 0.98, *p* = 0.952). A sex-specific effect was not significant (male: OR = 1.63, *p* = 0.314), with a low explanatory value (R^2^ = 0.016).

There was a similar trend for endurance in play: male horses tended to have higher odds (OR = 2.50, 95% CI: 0.96–6.56, *p* = 0.062), but without statistical significance. The R^2^ was 0.056, indicating a potential role of sex. Again, the higher odds indicate that male horses were more often attributed greater endurance.

In summary, male horses showed consistently but not significantly higher odds (OR between 1.6 and 2.5) in all three analyses, indicating a possible but unconfirmed sex-specific effect (Table 3).

### 3.4. Factors Affecting Injury Risk Perception

The logistic regression analysis examined the factors handicap, sex, age, and the number of horses owned or ridden in relation to the subjective assessment of injury risk. A significant association was found only for the players’ handicap (B = −0.078; *p* = 0.033). With increasing handicap value—and thus with greater playing strength and experience—players tended to assess the risk of injury for their horses as lower. The handicap groups ranging from −2 to 0 represented the majority of the sample (*n* = 89; 63.1%), whereas higher handicaps of +4 or above were only represented in isolated cases (*n* = 5; 3.5%).

Sex (male: *n* = 75, 52.4%; female: *n* = 68, 47.6%; *p* = 0.508), age (under 30 years: *n* = 51, 35.2%; over 30 years: *n* = 94, 64.8%; *p* = 0.513), and the number of horses owned or ridden (*p* = 0.616) showed no significant effect on risk perception (Table 4).

## 4. Discussion

This study provides systematically collected data on the subjective assessment of injury risk and the health burden of polo horses from the perspective of active players. The focus was on correlations between player characteristics and lameness incidence, as well as sex-specific differences in performance assessment.

### 4.1. Risk Assessment—Experience Influences Perception

A key result concerns the players’ perception of risk. Only the handicap, which reflects both playing experience and level of play, showed a significant influence on the assessment of injury risk. Players with a higher handicap systematically rated the risk as lower. There may be several explanations for this finding. First, habituation effects may occur, leading to an underestimation of danger due to routine [22]. Second, it is conceivable that more experienced players encounter lower risks because of better management practices or a more selective choice of horses [23]. Third, riders with higher handicaps are generally more skilled or experienced and are therefore better able to recognize potential hazards and avoid risky situations. Due to the small case numbers in the higher handicap groups (≥+5), however, the explanatory power of these results is limited, and the interpretation of regression coefficients in these subgroups should be made with caution.

A comparable observation can be found in equestrian sports. One study has shown that experienced riders consistently assess the risk of falls and injuries as lower than less experienced riders, even when objective risk factors remain similar. This suggests that greater skill and experience can influence subjective risk assessment in horse sports [1].

These findings align with the results of our study, in which more experienced polo players rated the risk of injury to their horses as lower. Although the cited study focuses on the riders’ own risk of injury rather than that of the horses, it illustrates that differences in risk perception depend on the experience of the riders.

A comparable observation also can be found in unrelated fields, for example, in a study on risk perception of various biotechnological applications: there too, laypeople consistently rated risks higher than the experts surveyed [24].

The fact that gender, age and number of horses had no significant influence on risk perception indicates a largely homogeneous assessment within these groups. The results suggest that experience in the game—more than demographic characteristics—shapes risk awareness.

### 4.2. Lameness—Objective Factors Prevail

Regarding the occurrence of lameness, there was a significant influence of horse age and breed. The risk of lameness increases significantly with age, consistent with existing literature and pointing to age-related degeneration during exercise [25,26]. Particularly striking was the significantly increased risk of lameness in horses of the “Polo Argentino/Thoroughbred” cross. One possible explanation could be that, although these crosses inherit athletic traits such as speed and agility from the Thoroughbred, they are not sufficiently compatible with the high physical demands and resilience required in polo. It should be noted, however, that the category “Polo Argentino” does not preclude the inclusion of other bloodlines, such as Thoroughbreds, in the breeding history, meaning there is no strict genetic separation. As there are no comparable studies available and the number of cases in this subgroup was very small, this result is not widely applicable.

It would therefore be advisable to conduct an objective study in this area with clinically confirmed lameness diagnoses.

Husbandry [extended stabling vs. year-round free stabling] had no significant influence on lameness. This partly contradicts previous assumptions about the preventive effect of free stabling [4,27,28]. It is possible that other stress factors, such as transport, tournament frequency, or ground conditions, are more relevant in polo than the basic type of housing. Studies with standardized recording of husbandry and stress factors are required to clarify this contradiction. The assumption in other studies that stallions and geldings are exposed to a higher risk of lameness compared to mares is also not confirmed by these data [29,30].

### 4.3. Performance Evaluation—Sex Differences

The ordinal regression analyses on willingness to perform, ambition, and endurance revealed no statistically significant differences between male and female polo horses. However, the consistent trend of slightly higher ratings in male horses, particularly in endurance, may indicate underlying physiological or behavioural mechanisms. Male horses could, for example, exhibit subtle hormonal or musculoskeletal differences that enhance stamina or responsiveness under exercise, which were not captured at a statistically significant level in this sample. That should be investigated further in future studies, for example, using objective performance data or blinded assessments. It must be noted that the three performance dimensions, willingness to perform, ambition in play, and endurance during the game, represent only the players’ subjective perceptions when no examples of horse characteristics corresponding to the ratings from low to very high were provided. The ambiguity of these definitions should be acknowledged as a limitation.

The results regarding horse sex appear contradictory. While most players reported that their best-performing and healthiest horses were mares, the regression analyses indicated a consistent, though not statistically significant, trend toward greater willingness, ambition, and endurance in male horses. This discrepancy may reflect both the traditional preference for mares in polo and the limited explanatory power of the regression models, which were based on small subgroups. 

In a comparable study from 2020 that examined various parameters influencing horse performance in British eventing competitions, the top performance of mares was lower than that of geldings and stallions at all levels [31]. However, the high subjective appreciation of female horses [73.8% of “best performing horse” = mare] in this study suggests that mares play a central role in polo—possibly due to their temperament, reliability, and dominance, as described in a 2019 study [32].

### 4.4. Causes of Injury—Focus on Ground Conditions

The most frequently cited cause of injury was poor ground conditions, which aligns with findings from other equestrian sports. Hard or uneven surfaces have been shown to increase the risk of tendon and joint damage [33,34]. This is also consistent with the survey-based study by Inness and Morgan, in which hard ground was identified as the most important risk factor [4]. The high frequency with which this factor was mentioned suggests that players directly associate it with health risks. Other cited causes, such as collisions, ball strikes, and falls, highlight the high physical demands and external influences in polo that have not yet been adequately investigated.

### 4.5. Methodological Reflection and Limitations

Despite the detailed data collected, the significance of this study is limited by certain methodological restrictions. Although internet-based data collection offers broad reach and anonymity, it is susceptible to self-selection bias and response distortion—especially in subjective assessments. The small number of cases in certain subgroups [e.g., Thoroughbreds] makes generalizing the results difficult. The decision to survey only active players was methodologically sound but limits the perspective to a specific subset of the polo community. No verification was conducted to confirm whether the participants were truly active polo players or to prevent multiple submissions of the survey, which constitutes a limitation of this study. Another limitation of this study is the uncontrolled distribution of the survey. Dissemination via social media led to participation from multiple countries without any control over the sample. The distribution of participants’ countries of origin should therefore be considered purely descriptive and must be considered when comparing the data. Nevertheless, the results provide a solid starting point for further research.

### 4.6. Implications for Practice, Ethics and Research

The results underline the need for systematic recording of health risks in polo. This is particularly relevant for investigating potential breed-specific differences and age-related factors as suggested by player perceptions, as well as for understanding players’ subjective risk awareness. Targeted prevention strategies could be developed on the basis of these findings—for example, through specific training adjustments or consideration of breeding criteria. The management of older polo horses could also be sensibly adapted in this way [35].

At the same time, the data show that greater cooperation between international professional associations, science, and the veterinary profession is necessary to establish uniform health standards. A standardized lameness protocol or a centralized injury tracking system—potentially supplemented by AI-based evaluations—would be conceivable [36,37]. Against the backdrop of growing ethical debates about the legitimacy of equestrian sports, well-founded risk research is becoming increasingly relevant to society [38,39]. Transparent communication of health risks could contribute to the long-term social acceptance of the sport or help initiate necessary reforms.

## 5. Conclusions

This study provides nuanced insights into risk factors and perception patterns in polo. While handicap influences risk assessment, owners perceived objective factors such as horse age and breed to have a significant impact on lameness risk. Future studies should include objective diagnoses, larger sample sizes, and longitudinal designs to further refine these findings and contribute to improved horse welfare.

## Figures and Tables

**Table 1:** Descriptive statistics on the sample and key variables of the survey (*n* = 145). Values given in percentage or as means and quartiles.

Category	Feature	Value/Distribution
Gender of players	Female	47.6%
	Male	52.4%
Age of players	Under 30 years	35.2%
	30 years and older	64.8%
Polo experience (in years)	Mean	13.38
	1st quartile	4.00
	3rd quartile	17.75
Handicap	Mean	0.28 (scale −2 to +10)
Horses owned or in training	Median	6 horses
	Range	0–101 horses
	1st quartile	3.00 horses
	3rd quartile	10 horses
Highest performing horse	Female	74.8%
	Male	25.2%
Most robust horse	Female	61.8%
	Male	38.2%
Optimal number of horse changes per chukker	No change	25.4%
	One change	45.9%
	Two changes	11.5%
	Individual regulation	17.2%
Evaluation of chukker duration (7:30 min)	Very good	53.4%
	Good	23.7%
	Acceptable	9.2%
	Less good	9.2%
	Not good at all	3.8%
Preferred mallet length	Size 49	0.7%
	Size 50	6.3%
	Size 51	14.6%
	Size 52	62.5%
	Size 53	16.0%
Most common causes of injury	Poor ground conditions	58.6%
	Collisions	24.1%
	Hits (ball/mallet)	20.0%
	Falls	14.5%
Injury risk assessment	High	15.4%
	Elevated	56.9%
	Low	26.9%
	No risk	0.8%

**Table 2:** Results of the logistic regression analysis on the risk of lameness in polo horses (*n* = 145). Odds ratios (OR) with 95% confidence intervals (CI) and *p*-values are shown. A significant influence is present at *p* < 0.05.

Factor	OR	95% CI	*p*-Value
Polo player: Experience (years)	1.06	0.98–1.15	0.152
Polo player: Gender	2.78	0.48–16.03	0.253
Polo player: Age	0.28	0.06–1.22	0.089
Husbandry: year-round free stabling	1.11	0.18–6.72	0.913
Horse breed: Polo Argentino × Thoroughbred	9.96	1.16–85.47	0.036
Horse breed: Thoroughbred	2.51	0.42–15.11	0.316
Horse: Sex (female)	1.39	0.32–5.98	0.663
Horse: Age (per year)	1.20	1.02–1.40	0.030

**Table 3:** Odds ratios (OR), 95% confidence intervals (CI) and *p*-values for the influence of willingness to perform, ambition, endurance and sex of the horse on the investigated event. The reference categories are “partly/partly” for the threshold variables and “female” for sex. A significant influence is present at *p* < 0.05.

Category	Threshold	OR	95% CI Lower	95% CI Upper	*p*-Value
Willingness to perform	very high	2.43	1.33	4.43	0.004
	high	35.15	10.09	122.29	<0.001
Sex = male		2.52	0.94	6.75	0.067
Sex = female (Ref.)		1.00	–	–	–
Ambition in play	very high	0.98	0.57	1.69	0.952
	high	13.45	5.41	33.42	<0.001
Sex = male		1.63	0.63	4.24	0.314
Sex = female (Ref.)		1.00	–	–	–
Stamina in play	very high	0.98	0.56	1.68	0.928
	high	10.28	4.52	23.42	<0.001
Sex = male		2.50	0.96	6.56	0.062
Sex = female (Ref.)		1.00	–	–	–

**Table 4:** Results of the analysis of variance to assess the risk of injury for horses in polo (*n* = 145). Unstandardized regression coefficients (B) and *p*-values are shown. A significant influence is present at *p* < 0.05.

Predictor	B	*p*-Value
(Constant)	3.292	<0.001
Player’s handicap	−0.078	0.033
Player’s gender	−0.088	0.508
Number of horses owned or ridden	0.002	0.616
Player’s age	−0.083	0.513

## Data Availability

The datasets presented in this article are not readily available because the data are part of an ongoing study. Requests to access the datasets should be directed to the corresponding author.

## Abbreviations

The following abbreviations are used in this manuscript:

CI	Confidence interval
OR	Odds ratio
